# ‘A false sense of security’? Understanding the role of the HPV vaccine on future cervical screening behaviour: a qualitative study of UK parents and girls of vaccination age

**DOI:** 10.1258/jms.2011.010148

**Published:** 2011-03

**Authors:** Lorna Henderson, Alison Clements, Sarah Damery, Clare Wilkinson, Joan Austoker, Sue Wilson

**Affiliations:** Cancer Research UK Primary Care Education Research Group, University of Oxford, UK; Cancer Research UK Primary Care Education Research Group, University of Oxford, UK; Primary Care Clinical Sciences, University of Birmingham, UK; Cardiff University School of Medicine, Wrexham, UK; Cancer Research UK Primary Care Education Research Group, University of Oxford, UK; Primary Care Clinical Sciences, University of Birmingham, UK

## Abstract

**Objectives:**

The UK Human Papillomavirus (HPV) vaccination programme was introduced in 2008 for girls aged 12–13. The vaccine offers protection against HPV types 16 and 18, which together cause about 70% of cervical cancers. Vaccinated girls will receive future invitations to the NHS Cervical Screening Programme, to prevent cancers associated with HPV types not included in the vaccine, and in case of prior infection with HPV 16 or 18. Little is known about parents' and girls' understandings of the protection offered by the vaccine, or the need for future screening.

**Design:**

Qualitative interviews with twenty-six parents, and nine girls aged 12–13 who were offered HPV vaccination through a Primary Care Trust (PCT) in the South-east of England, UK.

**Setting:**

Thirty-nine schools, and four general practices.

**Results:**

Uncertainty about the level of protection offered by the HPV vaccine was evident among parents, and to a lesser extent among vaccination-aged girls. There was a lack of understanding among parents and girls that cervical screening would be required irrespective of vaccination status; some parental decisions to accept the vaccine were made on the misunderstanding that vaccination provided complete protection against cervical cancer.

**Conclusions:**

Sufficient awareness of the issues related to screening is necessary for informed decision-making about whether or not to accept the HPV vaccine. Clearer information is needed concerning the incomplete protection offered by the vaccine, and that cervical screening will still be required. Future invitations for cervical screening should stress the necessity to attend regardless of HPV vaccination status, to ensure that high levels of prevention of cervical cancer through screening are maintained.

## INTRODUCTION

The Human Papillomavirus (HPV) vaccination programme was introduced in the UK in 2008 for all girls aged 12–13.^1^ A three-year ‘catch-up’ programme was also introduced in September 2008, for girls aged 14–17.^2^ The HPV vaccine used in the UK, Cervarix®, does not offer complete protection against all cervical cancers, but against the most common high-risk HPV types 16 and 18, which cause approximately 70% of cases of cervical cancer.^1,3^ Previous research suggests that parents' views on vaccinations play a pivotal role in whether or not their children are vaccinated^4,5^, and in the UK parental consent is required to vaccinate girls aged under sixteen years. However, parental perspectives on the HPV vaccination, and the rationale on which decisions are made to accept or decline the vaccine are unclear, as research to date has largely examined planned rather than actual behaviour.^6,7^

It is important that parents and girls make informed decisions about accepting the HPV vaccine based on accurate understandings of the advantages and disadvantages that vaccination offers, particularly the level of protection it provides against cervical cancer.^8^ Previous studies have reported low levels of knowledge and understanding of HPV, and the HPV vaccine, among parents and vaccinated girls^9,10,11^, and have shown that decisions are often made without sufficient awareness of relevant issues.^12^

The NHS Cervical Screening Programme will continue to play an important role for vaccinated girls by protecting them against cervical cancers caused by the high-risk HPV types not included in the vaccine, and to help prevent cervical cancer in those already infected with HPV.^2,13–15^ There is concern, however, that not all parents and girls who accept the offer of the HPV vaccine will be aware of these issues, and may assume they are fully protected from cervical cancer.^8^ Such a misconception among vaccinated girls could lead to a reduction in future participation in cervical screening, and decreased protection against cervical cancer as a consequence.

The study we report here is one component of a larger project which aims to develop evidence-based core HPV messages, relevant to the new testing and vaccination programmes, through the synthesis of systematic reviews, a series of qualitative interviews, and public surveys.^16^ The interview series included an exploration of the information needs of the first cohort of vaccination-aged girls and their parents, in relation to the decision to accept or decline the HPV vaccine. Uncertainties emerged surrounding the relationship between the level of protection the HPV vaccine provides against cervical cancer, and the need for future cervical screening. These issues were explored during subsequent interviews, and it is these exploratory findings that are reported in this paper.

## METHODS

### Participants and procedures

Qualitative research aims to achieve detailed levels of understanding through in-depth interviews and systematic analysis, and is the method of choice when little is known about the issues under investigation. We adopted a qualitative methodology in this study as an individualized and discursive approach is well suited to the exploration of issues such as participant awareness, information needs and understandings in relation to acceptance of the HPV vaccine, and the investigation of individual values and barriers to uptake or decline in the real life setting.

Qualitative data were collected from 37 parents whose 12–13-year-old daughters had been offered the HPV vaccine, and 44 girls aged 12–13 years who had been offered the HPV vaccine during the first wave of the HPV programme (September 2008) within one Primary Care Trust (PCT) in the South-east of England (Table 1).

**Table 1:** Participant characteristics

Characteristics	*n* = 81	Participants who commented on cervical screening issues: parents *n* = 26; girls *n* = 9
**Ethnicity**		
White British	81	35
**Declined vaccination**		
Mothers interviewed	20	10
Mother and father interviewed	2	2
Girls interviewed	14	7
**Accepted vaccination**		
Mothers interviewed	15	14
Girls interviewed	30	2

There were three key recruitment strategies. First, parents who gave consent for their daughters to be vaccinated were recruited via postal invitations sent from four general practices; all were group practices, two rural, one inner-city and one urban. Second, girls who received the HPV vaccine were recruited separately via postal invitations from two state comprehensive schools. Third, girls, and the parents of the girls who were not vaccinated were identified by the school nurse teams within thirty-nine schools and recruited via postal invitations; included were city and rural state comprehensive, independent, faith-based, and mixed and single sex schools. All potential participants were sent an invitation pack, which contained an introductory letter, information sheet, consent form, reply slip and stamped addressed envelope. Parental agreement for their daughters to participate in a research interview was required. Parents and girls were offered a gift voucher (£20 and £10 respectively) to thank them for their participation in the study.

One of the authors (LH) interviewed the parents between July 2009 and June 2010. The majority of interviews were conducted in the parents' home, and with the exception of two interviews which took place with both parents, were conducted solely with the mothers. The girls were interviewed by AC between October 2008 and April 2010. Thirty-eight girls chose to be interviewed in their own homes, two with their mothers present; and six girls chose small group discussions at their school.

Semi-structured interview topic guides were used to explore parents' and girls' reasons for accepting or declining the HPV vaccination. The topic guide provided a flexible set of content areas (including understandings of the purpose of the vaccine, the relationship between HPV and cervical cancer, the decision-making process, reasons for uptake and non-uptake, information needs, and future vaccination intentions) to direct the interview process, while allowing the participants to raise areas of relevance to them. Cervical screening as an area of uncertainty and relevance to the decision-making process originated from the parents, and, as the study progressed, subsequent interviews explored the understandings of the HPV vaccine in relation to cervical screening. The interviews lasted thirty minutes to one hour and were digitally audio-taped, transcribed verbatim, and anonymized. Ethical approval was granted by the Research Ethics Committee for Wales and written informed consent was obtained from the participants.

### Data analysis

The transcripts were reviewed to identify instances when parents or girls discussed cervical screening. Data was retrieved from twenty-six parent, and nine girl interviews (Table 1). From analysis of these data, themes emerged surrounding the level of protection offered by the HPV vaccine, the need for future cervical screening, and decision-making in the context of awareness of the need for future screening. A thematic analysis was combined with constant comparison of the data.^17,18^ The interviews were compared by selecting text which described similar or opposing experiences, both between interviews as well as in the context of each interview. A qualitative software package was used to help with the management of the data.^19^ LH and AC regularly discussed the coding and interpretation of the data to ensure a deep understanding, and to limit inconsistencies in the analytic process.

## RESULTS

The findings are presented around the three key themes which emerged during the analysis of parents' and girls' data, where cervical screening was discussed.

### 1. The level of protection offered by the HPV vaccine

The interviews revealed a range of understandings among parents about the level of protection the HPV vaccine offers against cervical cancer. At the time of making the decision as to whether or not their daughters should have the HPV vaccine, some parents believed the vaccine offered complete protection against cervical cancer.

‘Well it's sort of like a vaccine against cervical cancer: that's what it seemed to come across as … in the press they seemed to try and make you think this is a cure … if they had these injections (they) won't have to worry about cervical cancer at all. It would be totally almost unheard of if you, if everybody had the injections.’ (ID:W9 – mother who consented to have her daughter vaccinated.)

Other parents were at the opposite end of the spectrum, believing that the HPV vaccine offered only minimal protection against the disease.

‘At the end of the day it's only against one form of cervical cancer isn't it? It's only a minor prevention really.’ (ID:07 – mother who declined to have her daughter vaccinated.)

A number of parents were well informed, and aware of the limitations of the vaccine in the level of protection it afforded.

‘I've had abnormal cells myself so I am aware of that (how cervical cancer develops) and I do get screened very regularly, so I was aware that this is an anti-cervical cancer vaccine, but I was also aware that it doesn't protect against all of the viruses.’ (ID:NP02 – mother who declined to have her daughter vaccinated.)

Overall, the girls interviewed tended to have a greater understanding of the level of protection offered by the HPV vaccine, and realized that by completing the course of HPV vaccinations they would have a high, but not complete level of protection against developing cervical cancer.

‘All I really know about how it will prevent me from getting cancer is it will reduce the risk of it by seventy percent – and that it's a lot better than having cancer.’ (ID:21 – 12-year-old girl who received the HPV vaccine.)

### 2. Decision-making based on future cervical screening

It became clear that some parents had made the decision about whether their daughter would receive the HPV vaccine based on misconceptions about the need for cervical screening in the future. Several parents had believed that the vaccine would eliminate the need for cervical screening. The following mother perceived this to be a key benefit of being vaccinated, and had made her decision accordingly.

‘It sounded very positive! (the HPV vaccine) … If it meant that people didn't have to have smear tests when they were older that was great! … I don't like smear tests!’ (ID:W10 – mother who consented to have her daughter vaccinated.)

Another mother, who was unaware that the HPV vaccine offered limited protection, had also made her decision based on an inaccurate understanding. At the beginning of the discussion she had described the vaccine as ‘a miracle’, but when the incomplete protection offered by the HPV vaccine was described by the interviewer, she was surprised.

‘I wasn't aware of that. So you still need belt and braces?’ (ID:W3 – mother who consented to have her daughter vaccinated.)

The issue of whether or not cervical screening would be needed in the future was not part of the decision-making process for all parents. Some had greater levels of understanding than others, and were aware that cervical screening would still be necessary, whether or not the course of HPV vaccinations had been completed. This awareness was greater among parents who decided against accepting the vaccine for their daughter, possibly reflecting the additional information sought about HPV and the vaccine by those in this group. Rather than being information-based, for a number of mothers this realization came as a logical extension to knowing the vaccine only offered partial protection.

‘Oh yes, you would still need to go for a smear I'm sure but I haven't had any information on that I don't think. But because you can … there's always the fluke types that people get illnesses even after they've had the jab, and there were other sorts of cervical cancer that aren't caused by HPV is what I've understood …’ (ID:NP01 – mother who declined to have her daughter vaccinated.)

At the point of making the decision several parents remained uncertain as to whether vaccinated girls would need to attend cervical screening or not.

‘In the future they still need cervical screening? I don't know if that is clear actually.’ (ID:07 – mother who consented to have her daughter vaccinated.)

The few girls who had been vaccinated and who were aware of the cervical screening programme, usually through their mother's participation, were generally unclear of their need to attend screening in the future. The confusion surrounding screening is evident from the following quotation, which describes the belief that cervical screening will not be compulsory, but available for those who feel concerned.

‘Even though you've had the injection … if you're still worried then they can say in there (the leaflet) “don't worry, have the injection but if you're still worried then you can have scans when you're older.” I think that would be quite good to put in there (the leaflet).’ (ID:54 – 12-year-old girl who received the HPV vaccine.)

### 3. Information needs in relation to the HPV vaccine and future cervical screening

Several parents did not recall having received any information about the need for vaccinated girls to attend cervical screening in the future. The Department of Health information leaflet, provided to parents and girls with the invitation for HPV vaccination, states that cervical screening will still be required for vaccinated girls: ‘*The vaccine does not protect against all of the other cancer-causing types, so it's vital that women still go for routine cervical screening tests when they are older’*.^20,21^ The following parent, who consented to have her daughter vaccinated on the premise that she would not need to undergo cervical screening, only became aware of this misunderstanding when she read the Department of Health ‘Arm against cervical cancer’ leaflet at the time of interview.

‘I think from reading this leaflet it sounds like you still do have that screening … I did think that actually (that screening would not be necessary), but I realize it's more about actually reducing the instances … it's not reducing the need to screen.’ (ID:W10 – mother who consented to have her daughter vaccinated.)

Among parents who discussed cervical screening in the interviews, there was dissatisfaction with the delivery of the information about the HPV vaccine, and many felt they needed to conduct their own independent research on the topic.

‘To my knowledge it's not advertised in school that they should get that (screening) done anyway, unless, at some point later we start getting leaflets to be aware of these things … I think a lot of the information is purely what you can find out as a family really.’ (ID:07 – mother who declined to have her daughter vaccinated.)

Overall there was a profound lack of awareness among the girls interviewed concerning the NHS Cervical Screening Programme, with few having heard of smear tests, or cervical screening. During one interview clarification was sought from one girl about whether she had received any information about the need to attend cervical screening in the future.

Interviewer: One of the messages about the vaccination is that cervical screening is still really important – to pick up that remaining 30% (of cancers) that you are still at risk of. But it doesn't sound as though anybody's said that to you?

Girl: No. (ID:51 – 12-year-old girl who received the HPV vaccine.)

Providing accurate and relevant information concerning the level of protection that the HPV vaccine offers is vital in ensuring that vaccinated girls realize the importance of cervical screening in offering protection against all cervical cancers. The following mother described the vaccine as offering a false sense of security, and the danger of vaccinated girls believing they are no longer at risk of developing cervical cancer.

‘It gives you a sense of security … a false sense of security! I wouldn't be surprised if in that age group the number of people going for smears drops off because you think: “Well I've got protection you know!” You think you're vaccinated against everything – it's hard to remember always that it's that thirty percent!’ (ID:NP02 – mother who declined to have her daughter vaccinated.)

## DISCUSSION

This exploratory study has revealed inconsistencies among parents' and daughters' understandings of the HPV vaccine in two key areas: the degree of protection which the HPV vaccine offers against cervical cancer, and the need to attend cervical screening in the future irrespective of HPV vaccination status. The study findings also illustrated that the girls tended to have a greater understanding than their mothers of the level of protection the HPV vaccine offers against cervical cancer, but were broadly unaware of the NHS Cervical Screening Programme.

Some parents had accepted the offer of vaccination based on the belief that their daughters would not need to attend cervical screening in the future. The need for future attendance is clearly stated in the Department of Health leaflets provided to girls, and the discussion sheets designed for parents and girls.^20,21^ That this key message is not being clearly communicated highlights the need for ongoing educational interventions, tailored to vaccinated girls and their parents, to clarify that they will not be completely protected from cervical cancer when vaccinated, therefore cervical screening at a later stage will remain important for the prevention of cervical cancer. Our finding that girls have little awareness of the NHS Cervical Screening Programme is unsurprising, given the age differences of girls involved in the vaccination programme, and those eligible for cervical screening. However, raising awareness of the role of screening in the prevention of cervical cancer among younger girls could be beneficial, but will certainly need to be reinforced at the time of invitation to screening, particularly in light of recent research highlighting the decline in the number of young women attending for cervical screening.^22^

Parents' lack of awareness as to whether vaccinated girls would still need to attend for cervical screening suggests that parents are not always making fully informed decisions concerning the HPV vaccine. Despite the availability of the information relating to the continued need to participate in cervical screening, it is clearly not being conveyed in a consistent way. If parents are to make informed decisions they require clearer information, perhaps delivered in an alternative format to leaflets. Highlighting the key messages on the vaccination consent form, clearly and simply, may be one way to overcome the problem of written information not always being referred to, or understood.

The findings of this study are supported by previous research, which notes the importance of educating members of the public to negate the danger that HPV vaccination programmes are viewed as a replacement for the existing NHS Cervical Screening Programme.^13,14^ One study, conducted within the Australian vaccination programme, similarly indicated that the understandings of adolescents at the time of vaccination were unlikely to promote future participation in cervical screening.^9^ Together these findings clearly establish that future invitations for screening will need to stress the importance of attendance regardless of HPV vaccination status to ensure the future uptake of cervical screening is not adversely affected, risking a possible increase in the number of cases of cervical cancer. The findings of this current study are also important because they suggest parents' understandings of the need for their vaccinated daughters to attend future screening may play an important role in their decision to accept or decline the HPV vaccine. This has not been a factor previously identified as important to parents in relation to decision-making about acceptance of the HPV vaccine.^23^

One limitation of the study is the relatively small number of parents and girls among the participants in this study with whom cervical screening in relation to the HPV vaccine was discussed. The importance of these (mis)understandings emerged at a relatively late stage in the interview process, when there was limited scope for further exploration. However, the insights that have been gained are important. The understandings that parents and vaccination-aged girls have of the role of cervical screening in preventing cancers caused by the HPV types not covered by the vaccine, and whether these understandings affect decision-making about acceptance of the vaccine, need further investigation, particularly if revised information materials are to be widely accessible, understandable and relevant.

A major strength of this study is that it explored the understandings and reactions of those making actual decisions about acceptance of the HPV vaccine, as opposed to the use of hypothetical scenarios and imagined reactions. We included in the sample both girls and parents who had, and had not, accepted the offer of the vaccine. It is now equally important to explore the uptake rates of cervical screening when the first cohort of girls offered the HPV vaccine becomes eligible for screening. While it will be several years before the younger cohort are invited, the older girls within the ‘catch-up’ programme are eligible now for screening within parts of the UK. An exploration of the impact of HPV vaccination on attendance at cervical screening would help to determine the relevance of these exploratory findings to practice.
